# *In vivo* Study of the Histone Chaperone Activity of Nucleolin by FRAP

**DOI:** 10.1155/2011/187624

**Published:** 2011-03-03

**Authors:** Xavier Gaume, Karine Monier, Françoise Argoul, Fabien Mongelard, Philippe Bouvet

**Affiliations:** ^1^Université de Lyon, Laboratoire Joliot-Curie, Centre National de la Recherche Scientifique (CNRS)/Ecole Normale Supérieure de Lyon, 69007 Lyon, France; ^2^Laboratoire de Physique, Centre National de la Recherche Scientifique (CNRS)/Ecole Normale Supérieure de Lyon, 69007 Lyon, France; ^3^Laboratoire de Biologie Moléculaire de la Cellule, Centre National de la Recherche Scientifique (CNRS)/Ecole Normale Supérieure de Lyon, 69007 Lyon, France

## Abstract

Nucleolin is a major nucleolar protein involved in various aspects of ribosome biogenesis such as regulation of polymerase I transcription, pre-RNA maturation, and ribosome assembly. Nucleolin is also present in the nucleoplasm suggesting that its functions are not restricted to nucleoli. Nucleolin possesses, *in vitro*, chromatin co-remodeler and histone chaperone activities which could explain numerous functions of nucleolin related to the regulation of gene expression. The goal of this report was to investigate the consequences of nucleolin depletion on the dynamics of histones in live cells. Changes in histone dynamics occurring in nucleolin silenced cells were measured by FRAP experiments on eGFP-tagged histones (H2B, H4, and macroH2A). We found that nuclear histone dynamics was impacted in nucleolin silenced cells; in particular we measured higher fluorescence recovery kinetics for macroH2A and H2B but not for H4. Interestingly, we showed that nucleolin depletion also impacted the dissociation constant rate of H2B and H4. Thus, in live cells, nucleolin could play a role in chromatin accessibility by its histone chaperone and co-remodeling activities.

## 1. Introduction

The nucleosome is the fundamental unit of chromatin. It is composed of 147 base pairs of DNA wrapped around a histone octamer containing a tetramer of H3-H4 and two dimers of H2A-H2B [1]. Core histones are small highly basic proteins (from 11 to 15 kDa). They possess a globular domain, containing the histone fold domain (HFD), involved in the formation of nucleosome core particles through interactions with other histones. As a general view, DNA compaction into nucleosomes protects and regulates DNA activities by preventing DNA interaction with nuclear factors. Higher-order chromatin compaction involves the linker histone H1. Nucleosome assembly could be divided in two steps (for review see [2]) where DNA is first wrapped around a histone H3-H4 tetramer before two histone H2A-H2B dimers can be added [3–6].

Chromatin specificity can be driven by PostTranslational Modifications (PTM), targeted to the flexible amino terminal tails of histones (phosphorylation, acetylation, methylation, poly-ADP-ribosylation, and monoubiquitylation), or by the incorporation of histone variants (for review see [7]) or linker histones (for review see [8]). Histone variants are nonallelic isoforms of conventional histones [7, 9]. Their incorporation into nucleosomes can affect chromatin structure and nucleosome stability. For instance, macroH2A is a vertebrate-specific variant of the H2A canonical histone. In addition to the histone fold domain and the amino terminal tail, macroH2A contains a large carboxy terminal domain [10]. This tail may interact with linker proteins such as histone H1 and HMG (High-Mobility Group) proteins. macroH2A is enriched on the inactive X chromosome in mammalian female cells [11] and macroH2A containing nucleosomes are supposed to be less mobile compared to H2A and may be resistant to active transcription [12–14].

Nucleosomes are key factors for Eukaryotic genome compaction and they form a polar barrier for several processes involving DNA [15]. Indeed, nucleosomal structure is not favorable for DNA accessibility during DNA transcription initiation and elongation by RNA polymerases, DNA replication and repair. For instance, *in vitro* transcription initiation and elongation on chromatin templates are less efficient in comparison with naked DNA [16, 17]. However, in cells, DNA accessibility is rendered locally possible thanks to the action of several factors (like chromatin remodeling complexes and histone chaperones) that can either move the nucleosome along the DNA, transiently destabilize the histone DNA contacts, or completely disassemble and evict the nucleosomal structure from DNA.

Histone chaperones promote equilibrium between nucleosome assembly and partial disassembly or total eviction during several nuclear processes such as DNA transcription, DNA replication and DNA repair. In addition to their role in histone deposition and eviction, histone chaperones are also involved in nuclear import of histones and in their storage when not assembled onto DNA. This storage function prevents histone aggregation and interaction with inappropriate proteins. To promote accurate positioning of histones and regular spacing between nucleosomes, histone chaperones act in coordination with remodeling factors [18].

Recently, a histone chaperone activity has been reported *in vitro* for nucleolin, a nucleolar protein [19]. Nucleolin (also known as C23) is one of the most abundant nonribosomal proteins of the nucleolus. This 77 kDa protein is highly conserved in vertebrates and analogous proteins can be found in plants and yeast [20]. The amino acid sequence of nucleolin comprises three main domains: 1/the N-terminal domain, composed of four acidic stretches, is the site of numerous phosphorylations by Casein-Kinase 2 (CK2) and Cyclin-dependent-kinase-1 (Cdk1); 2/the central domain, containing four RNA recognition motifs, controls rRNA processing; 3/the C-terminal domain, a Glycine-Arginine-rich region, is implicated in nucleolar localization of the protein [20]. Nucleolin functions include regulation of Polymerase I transcription, pre-rRNA maturation and folding, ribosome assembly, and nucleocytoplasmic transport [20, 21]. However, nucleolin localization is not restricted to nucleoli. Indeed, nucleolin can be found in nonnucleolar nucleoplasm localizations, cytoplasmic granules, and at the cell membrane. We recently reported that nucleolin silencing by siRNA in human cells leads to numerous nuclear alterations (presence of micronuclei, multiple and large nuclei), cell growth reduction, accumulation in G2-phase, and increase of apoptosis [22]. Furthermore, abnormally high centrosome number has been found in silenced nucleolin cells [22], thus suggesting that nucleolin might play a role in the regulation of centrosome duplication. 

In their paper, Angelov et al. [19] demonstrated *in vitro* that nucleolin possesses a histone chaperone activity. Nucleolin binds directly to H2A-H2B dimers and facilitates their assembly into nucleosomes on naked DNA [19]. Using *in vitro *sliding assays, nucleolin was shown to act as a chromatin co-remodeler by increasing the efficiency of two chromatin remodelers SWI/SNF and ACF (ATP-dependent Chromatin assembly and remodeling Factor, a member of the ISWI family) on canonical nucleosomes. Interestingly, macroH2A variant nucleosomes can also be remodeled by SWI/SNF and ACF only in the presence of nucleolin [19]. 

Nucleolin has several common functions with the histone chaperone B23 (Nucleophosmin, NPM). Like nucleolin, B23 is a nucleolar protein associated with preribosomal particles and plays an important role in ribosome biogenesis. Interestingly, B23 and nucleolin knock-down have a quite similar phenotype. Depletion of B23 in HeLa cells by siRNA leads to distortion of nucleolar and nuclear structures, because of defects in microtubule polymerization and cytoskeletal structure [23]. Nucleolin's histone chaperone activity is also similar to that of the complex FACT [24]. Like FACT, nucleolin facilitates chromatin transcription elongation by promoting the removal of H2A-H2B dimers during transcription [24, 25]. 

So far, the chaperone activity of nucleolin has only been demonstrated *in vitro*. To investigate whether nucleolin can regulate the dynamics of core and variant histones *in vivo*, we analyzed whether the absence of nucleolin in live cells impacts the deposition of H2A-H2B and macroH2A-H2B dimers or H3-H4 tetramers onto chromatin. The dynamics of the GFP fusion proteins H2B-eGFP, macroH2A-eGFP and H4-eGFP stably expressed in wild type cells was investigated by FRAP (Fluorescence Recovery After Photobleaching) and compared to that of nucleolin-silenced cells. To better understand the consequences of nucleolin depletion on nucleosome destabilization and histone dynamics in live cells, a mathematical model was implemented to calculate histone dissociation rate constants (*k*_off_).

## 2. Results

### 2.1. Characterization of Histone-eGFP and Nucleolin-mCherry Double Stable Cell Lines for FRAP Experiments

Our goal was to perform FRAP experiments in HeLa cells stably expressing a histone-eGFP fusion protein (H4-eGFP, H2B-eGFP or macroH2A-eGFP) and the nucleolin-mCherry that was used to monitor in live cells the efficiency of nucleolin silencing.

**Figure 1:**
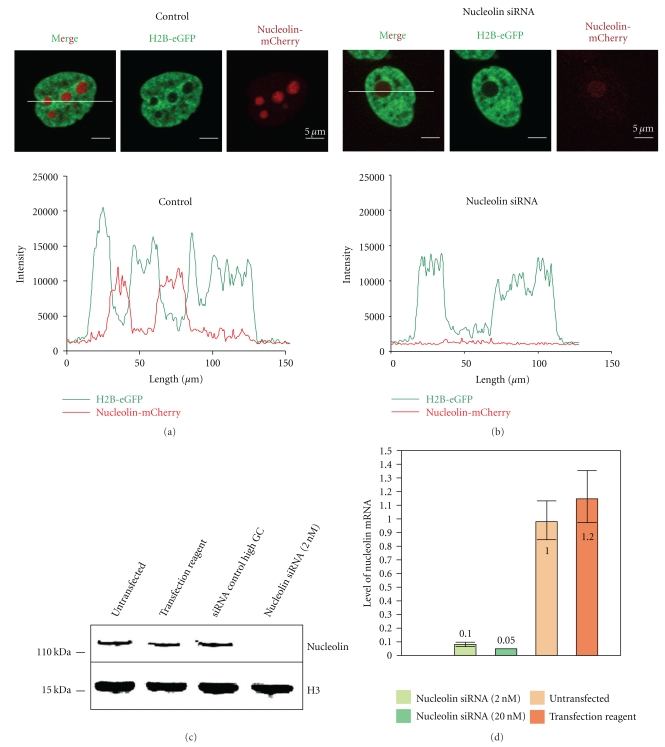
siRNA-mediated down-regulation of nucleolin. (a)-(b) Confocal section of live HeLa cells stably expressing nucleolin-mCherry (red) and the histone H2B-eGFP (green) visualized before (a) or after nucleolin silencing (b). Scale bars represent 5 *μ*m. H2B-eGFP and nucleolin-mCherry fluorescence intensity along the white lines are presented under the fluorescent images. (c) Western blot analysis of nucleolin in nonfluorescent HeLa cells: untransfected control cells, control cells transfected only with the transfection reagent, cells transfected with siRNA control high GC and siRNA against nucleolin-treated cells (mix siRNA #2 and #4 at 2 nM). Equal loading was verified using antihistone H3 antibody. (d) Quantitative RT-PCR analysis of nucleolin mRNA in nonfluorescent HeLa cells: untransfected control cells, control cells transfected only with the transfection reagent, siRNA against nucleolin-treated cells (mix siRNA #2 and #4 at 2 nM) and siRNA against nucleolin treated cells (mix siRNA #2 and #4 at 20 nM). Data were normalized with the amount of *β*-actin mRNA and the amount of mRNA in control cells.

In live HeLa cells, nucleolin-mCherry preferentially localizes to subnuclear regions exhibiting low fluorescence intensity (Figure 1(a), upper panel). The intensity profile of nucleolin-mCherry fluorescence (Figure 1(a), lower panel) was about five times higher in nucleoli of control cells than in nucleoplasm. H2B-eGFP fluorescence was not homogenous in the nucleus (Figure 1(a), upper panel). Along the intensity profile, the lowest H2B-eGFP nuclear fluorescence level colocalized with high nucleolin-mCherry fluorescence level (Figure 1(a), lower panel). Thus, H2B-eGFP fluorescence was generally low in nucleoli, except for small fluorescent granules present at the center of some nucleoli (data not shown). Likewise, in stable cell lines, H4-eGFP and macroH2A-eGFP were in majority excluded from nucleoli (data not shown). 

The siRNA transfection protocol was adapted in order to minimize cytotoxicity and off target effects (see Section 4). Nucleolin depletion was directly observed in live cells under a fluorescence microscope by visualizing nucleolin-mCherry. After nucleolin depletion, the majority of nucleolin-mCherry fluorescence disappeared except for small intensity spikes coming from small nucleolar dots (Figure 1(b) upper panel). This typical intensity profile of nucleolin-mCherry on nucleolin-depleted cells showed that the fluorescence level was roughly similar to that of background (Figure 1(b) lower panel). 

Quantitative analyses were performed to evaluate nucleolin silencing efficiency. Protein levels were analyzed by western blotting (Figure 1(c)) and mRNA levels by quantitative RT-PCR (Figure 1(d)). Four days after siRNA transfection, western blot analysis showed that nucleolin was no more detected in the cell extracts (Figure 1(c)). As controls, the transfection reagent alone or siRNA control highGC did not affect nucleolin protein level. Likewise, four days after siRNA transfection, only 5 to 10% of the nucleolin mRNA was still detected by quantitative RT-PCR in transfected cells compared to untransfected cells (Figure 1(d)). 

Based on these results, the upcoming photobleaching experiments were performed in nonnucleolar compartment four days after nucleolin siRNA transfection, in the three stable HeLa cell lines.

### 2.2. FRAP Experiments on Nuclear H2B-eGFP, H4-eGFP and MacroH2A-eGFP in Control Cells

**Figure 2:**
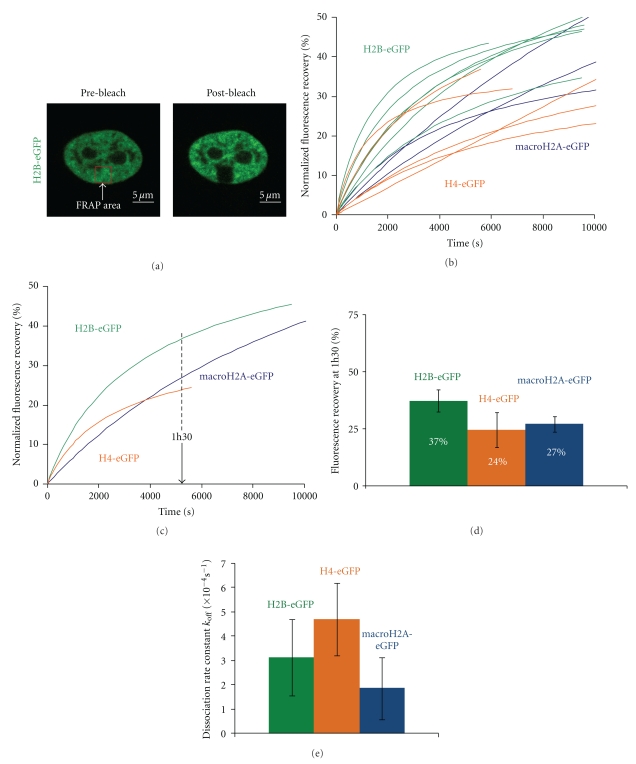
FRAP experiments on H2B-eGFP, H4-eGFP, and macroH2A-eGFP in nucleus of control untransfected cells. (a) Example of photobleaching on H2B-eGFP and nucleolin-mCherry stable cell line. Scale bars represent 5 *μ*m. (b) Normalized fluorescence recovery (%) of H2B-eGFP (green, 6 cells), H4-eGFP (orange, 5 cells) and macroH2A-eGFP (blue, 3 cells) in the nucleoplasm compartment. Each curve represents the fluorescence recovery of a single cell. (c) Average of the normalized fluorescence curves in (b) H2B-eGFP (green), H4-eGFP (orange) and macroH2A-eGFP (blue). (d) Comparison of the percentage of fluorescence recovery at 1h30 for H2B-eGFP (green), H4-eGFP (orange) and macroH2A-eGFP (blue). (e) Dissociation rate constant values *k*_off_ (s^−1^) of H2B-eGFP, H4-eGFP, and macroH2A-eGFP.

FRAP experiments were performed in HeLa cells stably expressing a histone-eGFP and the nucleolin-mCherry fusion proteins. In a first experiment, the H2B-eGFP, H4-eGFP and macroH2A-eGFP fluorescence recovery was compared after photobleaching a 13 *μ*m² square of the nucleus (Figure 2(a)). The normalized fluorescence recovery of H2B-eGFP, H4-eGFP, and macroH2A-eGFP in normal cells was plotted against time (Figure 2(b)) and each curve represents the normalized fluorescence recovery of an individual cell. In order to compare fluorescence recoveries, the average normalized fluorescence recovery was calculated for every eGFP-tagged histone as described in Section 4 (Figure 2(c)). One hour and half after photobleaching (5400 seconds), the normalized fluorescence recovery of H2B-eGFP was 37% (*σ* ^2^ = 4.9; *σ* ^2^ is the mean standard deviation: *σ* ^2^ = 〈*x* ^2^〉 − 〈*x*〉^2^), that of H4-eGFP was 24% (*σ* ^2^ = 7.61) and that of macroH2A-eGFP was 27% (*σ* ^2^ = 3.32) (Figure 2(d)).

The fluorescence recovery of H2B-eGFP at one hour and half was therefore faster than that of H4-eGFP showing that recovery rates obtained for core histones belonging to the dimer are faster than those belonging to the tetramer. This is in agreement with previous data from the literature [26]. Likewise, we found that fluorescence recovery of the histone variant macroH2A-eGFP was slower than the canonical histone H2B-eGFP. This slower recovery rate for macroH2A-eGFP compared to H2B-eGFP is consistent with its association with less active chromatin states.

### 2.3. Investigation of the Histone Dissociation Rate Constant with a Mathematical Model

What does imply a faster fluorescence recovery rate for the kinetics of assembly and disassembly of the core histones to the nucleosomal matrix? To address this question a mathematical model was implemented to determine the dissociation rate constant (*k*_off_) of this equilibrium (see also Section 4 for a detailed description). The percentage of fluorescence recovery against time depends on both the proportion of nucleosomes that are available for histone exchange and the dissociation velocity. In order to separate these two processes, a mathematical model (reaction and diffusion model) can be applied to the fluorescence recovery kinetics [27].

This model leads to reaction-diffusion equations considering freely diffusing proteins (histones) that can bind to a stationary structure (DNA). In a useful approximation, fluorescence recovery consists of a diffusional term in the first phase of recovery and a binding term dominating in the second phase. As the diffusion time of a free protein is very quick [28], the diffusion term can be ignored. Thus, the following equation can be applied to the FRAP recovery curves leading to the calculation of the dissociation rate constant *k*_off_:

(1) $$F\left(t\right)=C_{\text{eq}}\times \left(1-B\times e^{-k_{\text{off}}\times t}\right).$$

Using this mathematical model, we compared the dissociation rate constant *k*_off_ of H4-eGFP, H2B-eGFP, and macroH2A-eGFP in control cells (Figure 2(e)). Unexpectedly, the dissociation constant (*k*_off_) for H2B-eGFP does not appear to be higher than that of H4-eGFP, thus contrasting with the higher fluorescence recovery rate obtained for H2B-eGFP compared to H4-eGFP (compare Figures 2(d) and 2(e)). Thus, a faster fluorescence recovery rate does not necessarily imply a faster histone disassembly kinetics. Therefore, a third parameter needs to be introduced to explain these apparently contradictory results, which is the proportion of histone molecules involved in the transition from the bound state to the free state (noted *H*_*b*_ and *H*_*f*_ in Section 4). For instance, this implies that a sufficiently higher proportion of H2B dimer molecules than of H4 tetramers are transitioning from the bound state to the free state to counteract the slower *k*_off_ observed for H2B compared to H4 and give, in fine, a faster fluorescence recovery rate (see also Table 1, italic part). 

Comparison of H4 with macroH2A was also meaningful. Fluorescence recovery rate of macroH2A is comparable to that of H4, but the *k*_off_ is 2.5 times lower for macroH2A. This result is consistent with the fact that a smaller proportion of macroH2A-H2B dimers are dissociating compared to the dissociation of H3-H4 tetramers (Table 1, italic part). Comparison of H2B with macroH2A is the only case where a decreased fluorescence recovery rate correlates with a decreased K_off_, thereby assuming that a comparable proportion of H2B and macroH2A dimers within the FRAP region dissociate from chromatin (Table 1, italic part). Thus, macroH2A containing nucleosomes seem to be more resistant to histone dimer dissociation *in vivo *compared to H2B containing nucleosomes.

### 2.4. Investigation of Nucleolin Histone Chaperone Activity on Canonical Histones in Live Cells by FRAP

In order to assess the role of nucleolin on canonical histone dynamics, FRAP experiments have been performed on H2B-eGFP and H4-eGFP in cells that have been depleted in nucleolin using specific siRNA as previously described (Figure 1). 

**Figure 3:**
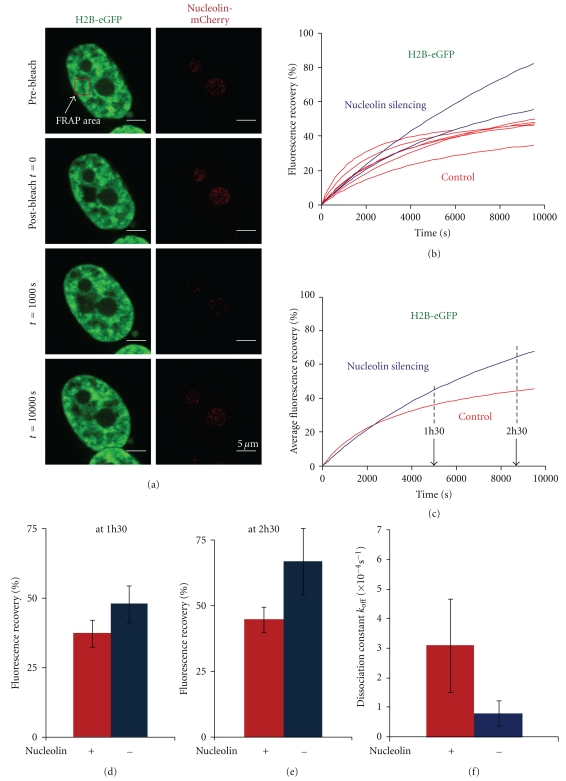
Nucleolin depletion leads to a faster H2B-eGFP fluorescence recovery kinetics. (a) Example of the fluorescence intensity recovery of H2B-eGFP histone in a nucleolin-depleted cell. Scale bars represent 5 *μ*m. (b) Normalized fluorescence recovery (%) of H2B-eGFP in the nucleoplasm of control-untransfected cells (red, 6 cells) and in cell transfected with siRNA against nucleolin (blue, 2 cells). Each curve represents the fluorescence recovery of a single cell. (c) Average of the normalized fluorescence curves in (b) control cell (red) and nucleolin depleted cells (blue). (d) Comparison of the percentage of fluorescence recovery at 1h30 for control cell (red) and nucleolin-depleted cells (blue). (e) Comparison of the percentage of fluorescence recovery at 2h30 for control cell (red) and nucleolin-depleted cells (blue). (f) Dissociation rate constant values *k*_off_ (s^−1^) of H2B-eGFP in control-untransfected (red) and nucleolin-depleted cells (blue).

After photobleaching, the fluorescence recovery of H2B-eGFP was followed in nucleolin-depleted cells (Figure 3(a)) and the normalized fluorescence recoveries of H2B-eGFP in individual control and nucleolin-depleted cells were plotted against time (Figure 3(b)), then the average normalized fluorescence recovery for each condition was calculated (Figure 3(c)). One hour and half after photobleaching (5400s), no significant difference in H2B-eGFP fluorescence recovery was noted between control and nucleolin-depleted cells (Figure 3(d)). However, 2h30 after photobleaching, a significant difference was observed, with 45% (*σ* ^2^ = 4.8) for control cells against 67% (*σ* ^2^ = 12.7) for silenced cells (Figure 3(e)). 

In terms of kinetics, the dissociation constant of H2A-H2B dimers in nucleolin silenced cells was four times slower than that in wild-type cells (Figure 3(f)). As described above, this implies that a higher number of nucleosomes are available for the dissociation of H2A-H2B dimers in nucleolin-silenced cells compared to wild-type cells, to explain the higher recovery rate observed in nucleolin-silenced cells (Figure 3(d) and Table 1, italic-bold part).

**Figure 4:**
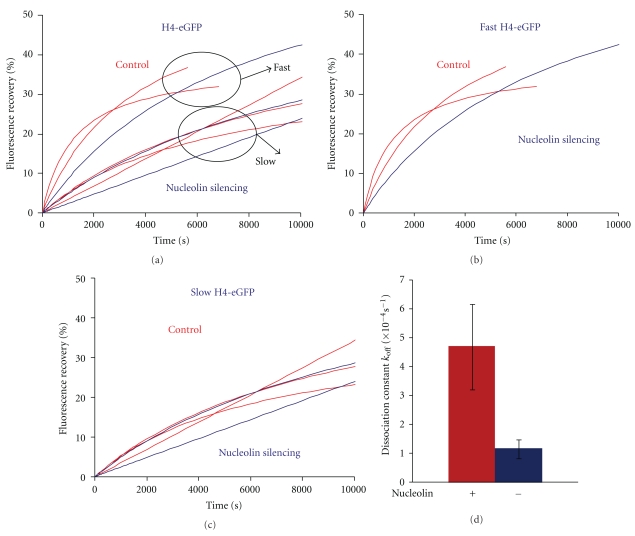
Nucleolin depletion does not impact H4-eGFP fluorescence recovery kinetics. (a) Normalized fluorescence recovery (%) of H4-eGFP in the nucleoplasm of control untransfected cells (red, 5 cells) and in cell transfected with siRNA against nucleolin (blue, 3 cells). Each curve represents the fluorescence recovery of a single cell. (b) Normalized fluorescence recovery (%) of H4-eGFP with a faster recovery kinetics: control untransfected cells (red, 2 cells) and siRNA against nucleolin transfected cells (blue, 1 cell). Each curve represents the fluorescence recovery of a single cell. (c) Normalized fluorescence recovery (%) of H4-eGFP with a slower recovery kinetics: control untransfected cells (red, 3 cells) and siRNA against nucleolin transfected cells (blue, 2 cells). Each curve represents the fluorescence recovery of a single cell. (d)/Dissociation rate constant values *k*_off_ (s^−1^) of H4-eGFP in control untransfected (red) and nucleolin depleted cells (blue).

The same experiment was performed with H4-eGFP (Figure 4). Cell-to-cell variations were observed for the fluorescence recovery curves of H4-eGFP, which can be subdivided into two populations (Figure 4(a)). Since these two populations are present for both control and nucleolin siRNA-treated cells and because cells showed similar efficiency of nucleolin-mCherry knock-down, it is unlikely that these two populations of cells result from different nucleolin levels due to uneven silencing efficiency. The population with faster recovery kinetics might correspond to cells in S-phase (Figure 4(b)). The population with slower recovery kinetics might correspond to cells belonging to gap phases (Figure 4(c)). With this group distinction, no significant difference was observed between control and nucleolin-depleted cells. In terms of kinetics, the dissociation constant of H3-H4 tetramers in nucleolin-silenced cells was four times slower than that in wild-type cells (Figure 4(d)). Since this difference in K_off_ does not translate into a smaller recovery rate, this implies that a higher proportion of H3-H4 tetramers dissociate in nucleolin-silenced cells compared to wild-type cells (Figure 4(d) and Table 1, italic-bold part).

In conclusion, nucleolin depletion leads to a higher fluorescence recovery rate for H2B-eGFP contrasting with a four times decrease of the dissociation constant of H2B-eGFP. For H4-eGFP, no difference in the fluorescence recovery rate was observed despite the four times lower dissociation constant in absence of nucleolin. These results imply that a higher proportion of histone molecules for both H2B-eGFP and H4-eGFP are mobilized from chromatin in nucleolin depleted cells. 

### 2.5. Investigation of Nucleolin Histone Chaperone Activity on MacroH2A Histone Variant, in Live Cells by FRAP

**Figure 5:**
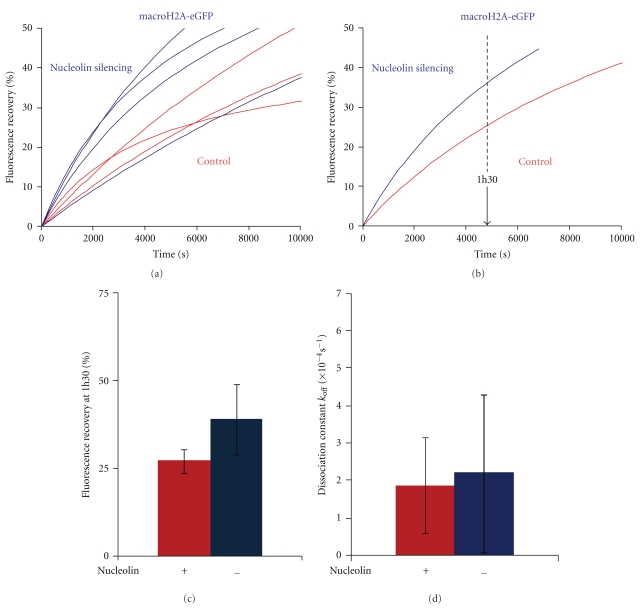
Nucleolin depletion leads to a faster macroH2A-eGFP fluorescence recovery kinetics. (a) Normalized fluorescence recovery (%) of macroH2A-eGFP in the nucleoplasm of control untransfected cells (red, 3 cells) and in cells transfected with siRNA against nucleolin (blue, 4 cells). Each curve represents the fluorescence recovery of a single cell. (b) Average of the normalized fluorescence curves in (a) control cell (red) and nucleolin-depleted cells (blue). (c) Comparison of the percentage of fluorescence recovery at 1h30 for control cell (red) and nucleolin-depleted cells (blue). (d) Dissociation rate constant values *k*_off_ (s^−1^) of macroH2A-eGFP in control untransfected (red) and nucleolin-depleted cells (blue).

Specific machineries are involved in the deposition of histone variants in chromatin. It is not known whether nucleolin is involved in this process, but previous experiments have shown that nucleolin is able to promote the remodeling of macroH2A variant nucleosomes [19]. These macroH2A nucleosomes could not be remodeled by SWI/SNF and ACF alone. These experiments suggest that the histone chaperone nucleolin is able to destabilize the macroH2A-H2B dimers or to modify the variant nucleosome structure to allow its remodeling by remodeling complexes. Thus, we wanted to investigate whether nucleolin could play a role in macroH2A dynamics *in-vivo*. To this end, using FRAP, we analyzed the changes in fluorescence recovery of macroH2A-eGFP in nucleolin depleted cells compared to normal cells (Figure 5). The fluorescence recovery of macroH2A-eGFP in control and nucleolin depleted cells was plotted against time (Figure 5(a)) and the average normalized fluorescence recovery for each condition was calculated (Figure 5(b)). One hour and half after photobleaching, the fluorescence recovery of control cells was 27% (*σ* ^2^ = 3.3), and that of nucleolin depleted cells was 39% (*σ* ^2^ = 10.06) (Figure 5(c)) indicating that after nucleolin depletion, the fluorescence recovery of macroH2A-eGFP is faster. In terms of kinetics, the dissociation constant derived from the mathematical model was not significantly different in nucleolin-silenced cells compared to wild-type cells. Thus the increase in fluorescence recovery observed in silenced cells cannot be explained by a difference in the dissociation constant. This implies that a higher proportion of macroH2A dimers are mobilized from chromatin in nucleolin-silenced cells.

## 3. Discussion

In this report, we investigated whether the histone chaperone activity of nucleolin, that has been described *in vitro* [19], could have some influence on histone dynamics in live cells. Thus, we compared histone dynamics by FRAP, in presence and absence of nucleolin. In control cells we first observed that H2B-eGFP (belonging to the H2A-H2B dimer) recovery rate was higher than H4-eGFP (belonging to the H3-H4 tetramer) and macroH2A-eGFP histone variant (Figure 2). In nucleolin-depleted cells, macroH2A-eGFP and H2B-eGFP presented a higher fluorescence recovery than in control cells (Figures 3 and 5), but no difference has been observed for H4-eGFP dynamics (Figure 4).

In order to investigate the molecular mechanism of these observations, we compared the dissociation rate constant of H4-eGFP, H2B-eGFP and macroH2A-eGFP in control and nucleolin-depleted cells. We found that nucleolin depletion leads to a lower dissociation rate constant of H2B-eGFP (Figure 3(f)) and H4-eGFP (Figure 4(d)) but not for the histone variant macroH2A-eGFP (Figure 5(d)). 

As a general view, FRAP analysis of histone dynamics reveals the kinetics of nucleosome assembly and disassembly. While nucleosomal structure is stable, different nuclear processes like DNA replication, transcription, and repair lead to histone eviction and replacement which is responsible for active histone dynamics. Thus, the cell cycle phase and the DNA metabolism processes are key factors in controlling histone dynamics. FRAP experiments allowed us to determine the dynamics of histones (histones in fusion with eGFP) by analyzing fluorescence recovery in the bleached region. The recovery rate may also depend on the chromatin state. Indeed, histone dynamics in heterochromatin and euchromatin could be different, as previously shown for H1 linker histone [28, 29], but not shown so far for core histones.

**Figure 6:**
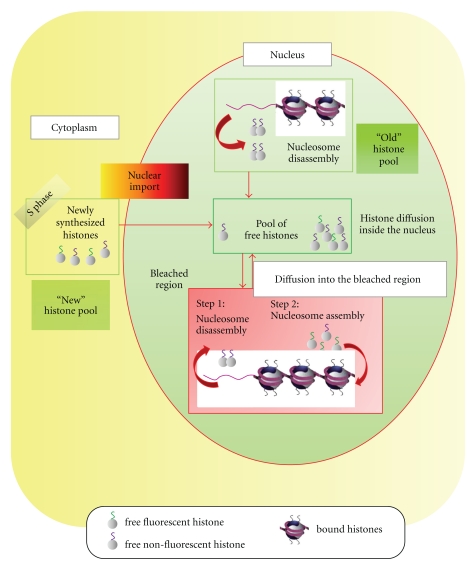
Schematic of histone exchange after photobleaching a subregion of a cell nucleus. Fluorescence recovery of histone-eGFP after photobleaching requires several molecular events. Timewise, the first step prior to observing histone fluorescence recovery is the disassembly of bleached histones from the chromatin in the FRAP region (step 1). The second step is their replacement by fluorescent histones coming from the pool of free nuclear histones (step 2), which can diffuse freely and rapidly, in the range of few seconds to cross the nucleus. The source of free nuclear histones comes from either “old” previously synthesized histones which have been disassembled from chromatin (“Old” histone pool) or from newly synthesized histones (“New” histone pool) imported in the nucleus. Thus, except in S-phase, when histone synthesis takes place, the pool of unbound histone is mostly nourished by the source of histone disassembly (“Old” histone pool). During S-phase, the pool of unbound nuclear histones will also be replenished by import of newly synthesized histones coming from the cytoplasm. After photobleaching, fluorescence recovery is the consequence of histones reassembly with free fluorescent histones coming from the nuclear pool of free histones.

Fluorescence recovery by histones-eGFP after photobleaching requires several molecular events (Figure 6). Time wise, the first step prior to observing histone fluorescence recovery is the disassembly of bleached histones from the chromatin in the FRAP region. The second step is their replacement by fluorescent histones coming from the pool of unbound nuclear histones, which can diffuse freely and rapidly, in the range of few seconds to cross the nucleus. The source of unbound nuclear histones comes from either “old” previously synthesized histones which have been disassembled from chromatin or from newly synthesized histones imported in the nucleus which make them available rapidly for *de novo* assembly. Thus, except in S-phase, when histone synthesis takes place, the pool of unbound histone is mostly nourished by the source of histone disassembly. During S-phase, the pool of unbound nuclear histones will also be replenished by import of newly synthesized histones coming from the cytoplasm (Figure 6). After photobleaching, fluorescence recovery is the consequence of histones reassembly with histones-GFP which are coming from outside of the bleached region. This involves relatively long-distance diffusion of histones and excludes that this fluorescence recovery is the consequence of nucleosome sliding which is rather a local phenomenon.

In our FRAP experiments we did not discriminate the cells according to their cell cycle phase. Thus, histone recovery kinetics measured histone replacement due to any of the previously described nuclear processes. This could explain the variations observed in the recovery rates for H4-eGFP (Figure 4(a)). Indeed, we found two different rates for H4-eGFP fluorescence recovery (Figures 4(b) and 4(c)). The faster recovery rate that is observed could correspond to the analysis of S-phase cells. During replication, histone turnover rate is expected to be faster than during gap phases due to the assembly of *de novo* synthesized histones imported from the cytoplasm as well as the spreading of old histones between the new and the old replicated DNA strands. The slower recovery rates could rather correspond to cells that belong to gap phases.

After nucleolin depletion, we found that H2B-eGFP fluorescence recovery was higher (Figure 3). However, the dissociation rate constant (*k*_off_) was lower than in control cells (Figure 3(f)). Thus, after nucleolin depletion, H2B exchanges slower but more H2Bs are available for exchange (explaining the higher fluorescence recovery). Therefore, nucleolin seems to be involved in both the H2A-H2B dimer dissociation velocity and in the proportion of nucleosome capable of exchanging H2A-H2B. The implication of nucleolin in the H2A-H2B dimer dissociation from nucleosomal template is consistent with previous *in vitro* analysis showing that the histone chaperone nucleolin is able to promote exchange of this dimer and the formation of hexasomes [19]. We also found that macroH2A fluorescence recovery was higher after nucleolin depletion (Figure 5). Interestingly, no significant differences are observed for the dissociation rate constant (Figure 5(d)). This indicates that nucleolin is probably not involved in the destabilization of macroH2A containing nucleosomes which would allow the macroH2A-H2B exchange. However, the higher fluorescence recovery rate in absence of nucleolin could be the consequence of an increased pool of macroH2A-nucleosomes available for histone exchange. No significant difference in H4 fluorescence recovery kinetics was observed after nucleolin silencing (Figure 4). However, H4 dissociation rate constant (*k*_off_) was lower than in control cells (Figure 4(d)), suggesting that nucleolin is involved in the rapid dissociation of the H3-H4 tetramer, and again, in absence of nucleolin, more nucleosomes are competent for the H3-H4 tetramer exchange to explain that the fluorescence recovery kinetic is not modified compared to control cells despite a four times lower dissociation rate constant.

Nucleolin knock-down by siRNA affects cell cycle progression and rDNA transcription [22, 30]. However, transcriptomic analysis of HeLa and human fibroblast cells depleted in nucleolin have shown that a small number of genes transcribed by RNA polymerase II are affected by the absence of nucleolin compared to normal cells (P. Bouvet, unpublished data). Therefore, depletion of nucleolin does not affect drastically the whole genome expression that could explain the changes in histone dynamics observed in this study. However, we cannot totally exclude that nucleolin silencing could modify the expression of a gene specifically involved in the regulation of chromatin dynamics that we have not yet identified in our transcriptomic analysis.

Previous experiments have demonstrated, *in vitro*, that nucleolin is an histone chaperone, able to promote the dissociation of H2A-H2B dimer and its exchange with other nucleosome templates [19]. Therefore, the changes of fluorescence recovery (histone dynamics) and of dissociation rate constant that we observed in live cells might be the first evidence of an *in-vivo* histone chaperone activity of nucleolin.

The observation that the dissociation rate constants for H2B and H4 are affected in absence of nucleolin is in good agreement with previous data showing that nucleolin is able to destabilize the nucleosomal structure and to promote the loss of H2A-H2B dimers [19]. Although nucleolin is able to promote the remodeling of macroH2A variant nucleosome [19] by remodeling complexes, nucleolin does not seem to be involved in the destabilization of the macroH2A-H2B dimer as the dissociation rate constant for macroH2A is not affected in absence of nucleolin.

In addition of being involved in the destabilization of nucleosome structure, this report also shows that in absence of nucleolin there is a higher proportion of nucleosome capable of exchanging histones. This may indicate that in absence of nucleolin, chromatin is more accessible to different nuclear factors. Indeed, microscopic analysis of nucleolin depleted cells, shows that these cells have bigger nuclei and nucleoli [22, 30] which could correspond to chromatin decondensation. Furthermore, there are now several reports showing that in addition to the organization of rDNA chromatin [22, 31] nucleolin is involved in chromatin condensation [32] and chromatin loop organization [33, 34]. Therefore, nucleolin could also be involved in the regulation of chromatin accessibility at relatively large scale compared to its effect on single nucleosome destabilization or to its co-remodeling activity with remodeling complex. The inhibition of nucleolin expression could therefore induce an important chromatin reorganization leading to an increase of the pool of nucleosomes capable of exchanging histones.

Histone chaperones are key factors for the dynamic organization of chromatin template and for the regulation of DNA metabolism such as DNA replication, repair and transcription. They could have some regulatory roles on specific genes (like rDNA genes for nucleolin) but also global function for the reorganization of chromatin in the cell nucleus. Fluorescence recovery changes observed in nucleolin-depleted cells could not only result from the action of chaperones on specific genes, but are rather the consequences of modified global chromatin organisation making nucleosomes more (or less) accessible to histone exchanges. Future genomewide analysis should bring new exciting data on the role of histone chaperones on global chromatin organization and gene regulation.

## 4. Materials and Methods

### 4.1. Cell Culture

HeLa cells were grown in CM-E medium defined as follows: *α*MEM medium containing Glutamax (PAA), complemented with 10% Fetal Calf Serum (FCS), 1% non essential amino acids, and 1% penicillin/streptomycin. Cells were maintained at 37°C in a 5% CO_2_-humidified incubator. Instead of trypsin, we used a mixture of collagenases referred to as accutase (PAA) for cell detachment.

### 4.2. Stable Cell Line Establishment

We transfected HeLa cells, stably expressing the nucleolin-mCherry protein (F. Mongelard and S. Storck), with H2B-eGFP-N1 (gift of T. Kanda), H4-eGFP-N1 (gift of O. Masui), or macro-H2A-eGFP (gift of P. O. Angrand) plasmids. Histone-tagged-GFP proteins were under the control of CMV promoter. 

One day before transfection, cells were plated at 3 × 10^5^ cells/dish in 6 well dishes. Cells were transfected in CM-E medium using jetPRIME DNA transfection reagent (Polyplus transfection). In order to get a collection of clones with various levels of fluorescence intensity, two parallel transfection conditions were performed with either 1 *μ*g of target DNA or with 0.1 *μ*g of target DNA + 0.9 *μ*g of pBlueScript plasmid (to keep the amount of DNA to 1*μ*g as recommended by jetPRIME manufacturer). One day after transfection, cells were plated in a 15 cm diameter culture dish. Depending of the efficiency of transfection, clones were isolated from either the first or second transfection condition.

In order to evaluate the optimal antibiotic concentration for clone selection, a killer test was performed. Nucleolin-mCherry HeLa cells were grown with different concentration of G418 and puromycin. The optimal antibiotic concentrations were chosen where 95% of cells were killed after 5 days. 

Clone selection was performed in CM-E containing 1mg/ml of G418 for H2B-eGFP-N1 and H4-eGFP-N1 or 0.3 *μ*g/ml of puromycin for macroH2A-eGFP. Ten to fifteen days after transfection, six double fluorescent clones were selected under a fluorescence microscope and then isolated. We insured that the proliferation rate was unchanged for clones and that they did not exhibit nuclear alterations. FRAP analyses are easier when the fluorescence is intense, especially for slow recovery rate. Thus, for FRAP experiments the brightest of the six selected clones was chosen for each of the three conditions. Nevertheless, we ensured that histone-GFP fluorescence was properly localized in the nucleus and was not too high to make sure that histone-GFP level was not higher than the level of endogenous histones.

### 4.3. Nucleolin Silencing by siRNA

A mixture of functional siRNAs specific for human nucleolin was used (described in [22]). Nucleolin siRNAs (Eurogentec) were reconstituted at a concentration of 100 nM and stored at −20°C  . We used a mixture of siRNA #4 (UUCUUUGACAGGCUCUUCCUU) and siRNA #2 (UCCAAGGUAACUUUAUUUCUU). As a siRNA control, we used stealth high GC siRNA (Invitrogen). Cells were transfected in a 6-well dish using siRNA at 2 nM final concentration. siRNAs were diluted in 200 *μ*l of Opti-MEM and plated in a well. 80 *μ*l of INTERFERin (Polyplus) diluted 1 : 10 in RNase-free water were added. After 10 min incubation, 2ml of medium containing 3 × 10^5^ cells were added. After 2 days, cells were detached and plated on 10 cm dishes or in 35 mm Ibidi dishes (*μ*-dish high iBIDI treat Biovalley) for microscopy analysis. FRAP experiments were performed 72 to 96 hours after transfection. Western-blot and quantitative PCR experiments performed on samples processed in parallel revealed a decrease of protein and a 90% decrease of mRNA level for nucleolin 72 hours after siRNA transfection.

### 4.4. Western Blot

Cells were collected 4 days after transfection in a lysis buffer containing 2% SDS, 10% glycerol, and 20%  *β*-mercapto-ethanol, for a final concentration of 1 × 10^4^ cells/*μ*l. 1 × 10^5^ cells were loaded onto a 10% SDS poly acrylamide gel electrophoresis. The proteins were then transferred to Protan membranes (Schlecheir and Schuell, Germany). Membranes were blocked in 5% milk and incubated with the primary antibodies for 1 hour in 1xPBS containing 1% milk. Nucleolin was detected with a mouse monoclonal antibody (4E2 Assay Designs/Immunogen: human nucleolin) (dilution 1/1000) and H3 with a rabbit polyclonal antibody (ab1791 Abcam) (dilution 1/2000). Secondary antibodies were also diluted in 1xPBS containing 1% milk for at least 2 hours. We used the IRDye 800CW conjugated Goat anti rabbit IgG and the IRDye 680 conjugated Goat anti mouse IgG (Licor Biosciences). Western blot imaging was performed with an Odyssey Infrared Imaging System (Licor Biosciences).

### 4.5. Quantitative PCR

Four days after siRNA transfection, cells were collected and stored in 1mL Trizol (Gibco BRL) at −20°C. Total RNAs were isolated according to the manufacturer procedure (Gibco BRL) and quantified using a Nanodrop (Thermo Scientific). Genomic DNA contamination was removed using DNase I RNase free (Fermentas). To perform the reverse transcription step, we used 100 ng of RNA. For each sample, we incubated the 100 ng of RNA with 20 U of RiboLock RNase Inhibitor (Fermentas), 0.95 mM of dNTP (Fermentas), 40 U of M-MuLV Reverse Transcriptase (Fermentas), and 0.2 *μ*g of Random hexamer primers (Fermentas). 1.5% of the cDNA resulting product was analysed by qPCR (Step one plus, Applied Biosystems), using a commercially available master mix containing Taq DNA polymerase and SYBR-Green I deoxyribonucleoside triphosphates (Roche). We carried out 40 cycles of denaturation (95°C for 15 seconds) annealing and extension (58°C for 1 minute). Cytoplasmic *β*-actin was analysed in parallel to each PCR and the resulting measurements were used as internal standards for normalization.

### 4.6. Live Cell Imaging and FRAP Experiments

Cells were plated on 35 mm Ibidi dishes (*μ*-dish high iBIDI treat Biovalley) two days after transfection. Control cells were untransfected and nucleolin depletion was carried out using the siRNA #2 + #4 at 2 nM. Live cell imaging was performed in CM-E medium without phenol red (to limit autofluorescence) in a thermo- and CO_2_-regulated atmosphere, with a FRAP spinning disk confocal microscope setup on a Leica-inverted microscope equipped with an EMCCD camera (Quantem, Universal Imaging Corp). The illumination system was composed of two laser benches, one with four laser lines (405, 491, 561, 635 nm) dedicated to acquisition, and one with 2 laser lines (405, 471 nm) dedicated to FRAP and photoactivation. Using the 491 and 561 laser lines and a 63x oil-immersion objective lens (NA = 1.4), a z-stack of 40 optical sections spaced of 0.6 *μ*m in the *z* direction was acquired (full chip of 512 × 512, bin1, 1pixel = 133 *μ*m), using a piezzostage. 

For each nucleus, a Region Of Interest (ROI) of 13 *μ*m² was bleached once with the 471 nm laser (diode Cobolt 25 mV, 20% of full power) for 5 milliseconds. 40 sections spaced of 0.6 *μ*m were acquired during a maximum of 6 hours. Five images were taken before bleaching. Then, for the first 5 min, one stack was acquired every 30 seconds, resulting in the collection of 10 stacks. During the next 24 min, one stack was acquired every 2 min, resulting in the collection of 12 stacks. Finally, we acquired one stack every 5 min until the end of the time lapse.

### 4.7. Quantification of Relative Fluorescence Intensity

Fluorescence intensity was measured using the ImageJ freeware on one optical section for each time point. During the several hour fluorescence recovery periods, cell displacement on the microscopic dish occurs on a regular basis, then resulting in X-Y and Z shifts of the nuclear position, often associated with nuclear morphology modifications. Thus, before fluorescence measurement, the position of the Region Of Interest (ROI) was adjusted when necessary in the X-Y and Z axis, for each z-stack.

The average intensity in the ROI before bleaching, immediately after bleaching (30 seconds) and during post bleaching was measured. Fluorescence intensity of the nucleus was also measured. The Relative Fluorescence Intensity (RFI) was calculated [35]:

(2) $$\text{RFI}=\frac{\text{ROI}\left(t\right)/\text{Nucleus}\left(t\right)}{\text{ROI}\left(t=0\right)/\text{Nucleus}\left(t=0\right)}.$$

ROI(*t*) is the average fluorescence intensity of the photobleached region at various time points after photobleaching, Nucleus(*t*) is the average fluorescence intensity of the entire nucleus at the corresponding time points, ROI(*t* = 0) is the average fluorescence intensity of the photobleached region before photobleaching, and Nucleus(*t* = 0) is the average fluorescence intensity of the entire nucleus before photobleaching.

Then, fluorescence recovery was plotted against time, and non-linear curve fitting was carried out [35] using the “curve fitting” function on ImageJ freeware, first to allow a quick survey of the different curves and compare their recovery efficiency:

(3) $$F\left(t\right)=a+\frac{\left(b-a\right)\times t}{t+c}.$$

In order to visually compare several experiments, the normalized fluorescence recovery was calculated from the equation of the fit and plotted as a percentage against time:

(4) $$F\left(t\right)=\frac{\left(\text{RFI}\left(t\right)-a\right)\times 100}{1-a}.$$

### 4.8. Dissociation Rate Constant Calculation

Assuming that the histones are either freely diffusing (Hf) or bound (Hb) to a steady structure (chromatin nucleosomes), the inclusion (adsorption) and the release (desorption) of a histone from chromatin can be described by a simple reaction:


(5)
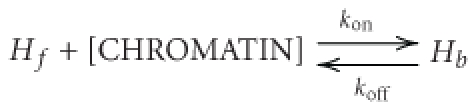

Assuming a linear diffusion for free histones (*D* being their diffusion coefficient), the model equations read:

(6) $$\begin{array}{c} \frac{\partial H_{f}}{\partial t}=D\nabla^{2}H_{f}-k_{\text{on}}H_{f}+k_{\text{off}}H_{b},\\ \frac{\partial H_{b}}{\partial t}=k_{\text{on}}H_{f}-k_{\text{off}}H_{b}. \end{array}$$

Fluorescence may be substituted for concentration since it is directly proportional to it. The initial time *t* = 0 corresponds to the end of bleaching, and the values of *H*_*f*_ and *H*_*b*_ prior to bleaching are related by the thermodynamics of the adsorption-desorption reaction:

(7) $$\frac{H_{f}^{\text{eq}}}{H_{b}^{\text{eq}}}=\frac{k_{\text{off}}}{k_{\text{on}}}.$$

Just after bleaching the fluorescent (bound and free) protein concentrations are depleted to the values: *H*_*f*_ and *H*_*b*_. We consider here the second phase of recovery, which occurs a few seconds after photobleaching, and we assume that the concentration of free protein in the bleached region is equivalent to that of the unbleached nucleus (the depleted bleached free protein reaches rapidly zero) [36]. Therefore the evolution equation for the depleted bound protein reads:

(8) $$\frac{\partial H_{b}}{\partial t}=-k_{\text{off}}H_{b},$$

which integrates readily to give the equation for the fluorescent bound protein:

(9) $$H_{b}=H_{b}^{\infty }\left[1-Be^{-k_{\text{off}}t}\right].$$

We have used this equation to fit the experimental curves. *B* is a parameter which characterizes the fraction of bleached bound proteins at *t* = 0:

(10) $$H_{b}^{0}=BH_{b}^{\infty }.$$

The nonlinear fitting of the experimental data was performed by a Levenberg-Marquardt algorithm under Matlab software.

## Figures and Tables

**Table 1:** Summary of the FRAP results obtained for recovery rates and dissociation constants (*K*_*off*_) extracted from Figures 2–5 for H2B, H4, and MacroH2A. The analysis was subdivided into 3 categories: control cells (in italic), silenced cells (bold), and comparison between silenced cells and control cells (italic-bold). In addition to the 2 parameters directly extracted from the results, implication of a third parameter was obvious to explain the observed recovery rates, the number of bleached histone molecules disassembled.

	H2B	H4	MacroH2A
*Relative recovery rate at 1h30 in control cells*	*faster (37%)*	*slower, but variable (24%)*	*slower (27%)*
*Relative * K_off_ * in control cells*	*medium*	*faster*	*slower*
*Implication for the number of bleached histone molecules disassembled in control cells*	*higher number compared to H4*	*lower number compared to macroH2A*	*compatible with same number for H2B*

**Recovery rate in silenced nucleolin cells at 1h30**	**faster**	**slower, but variable**	**faster**
K_off_ ** in silenced nucleolin cells compared to control cells**	**slower**	**slower in any case**	**faster**
**Implication for the number of bleached histone molecules disassembled in silenced cells**	**higher number compared to H4**	**smaller number compared to macroH2A**	**higher number compared to H2B**

***Recovery rate in silenced nucleolin cells compared to control cells***	***faster***	***identical when splitted into a fast and a slow population***	***faster***
K_off_ ***in silenced nucleolin cells compared to control cells***	***slower***	***slower in any case***	***identical***
***Implication for the number of bleached histone molecules disassembled in silenced cells***	***higher number compared to control***	***higher number compared to control***	***higher number compared to control***
